# Unveiling Sodium Diffusion Kinetics and Locking Mechanisms for High‐Performance CZTSSe Photovoltaics

**DOI:** 10.1002/advs.202504087

**Published:** 2025-05-23

**Authors:** Shuyu Li, Chaoran Li, Chu Liu, Jiachen Wu, Letu Siqin, Yuan Li, Guonan Cui, Yanchun Yang, Ruijian Liu, Hongmei Luan, Chengjun Zhu

**Affiliations:** ^1^ Inner Mongolia Key Laboratory of Semiconductor Photovoltaic Technology and Energy Materials Center for Quantum Physics and Technologie School of Physical Science and Technology Inner Mongolia University Hohhot Inner Mongolia 010021 China; ^2^ School of Physics and Electronic Information Inner Mongolia Normal University Hohhot Inner Mongolia 010022 China

**Keywords:** Cu_2_ZnSn(S,Se)_4_, interface passivation, Na diffusion, Na locking, solar Cells

## Abstract

This work unveils a diffusion‐kinetic modulation strategy that fundamentally redefines sodium management in kesterite photovoltaics, enabling spatially controlled Na sequestration within Cu_2_ZnSn(S,Se)_4_ (CZTSSe) absorber layers through a thermally engineered “Na‐locking” mechanism. By establishing critical correlations between post‐processing thermal protocols and alkali metal migration dynamics, how synchronized extension of sintering duration and rapid cooling termination creates a non‐equilibrium state that traps Na at strategic interfacial positions is demonstrated. This approach leverages Na's dual functionality as a crystallization promoter and defect passivator, driving concurrent improvements in crystallographic coherence and electronic uniformity. The optimized absorber architecture features laterally expanded grains with reduced boundary density and homogenized interfacial charge transport pathways, yielding the highest reported efficiency of 13.22% for Na‐doped CZTSSe solar cells to date, marked by synergistic enhancements in both *V_OC_* and *FF*. Crucially, this substrate‐derived Na regulation paradigm outperforms conventional extrinsic doping methods through its self‐limiting diffusion characteristics, ensuring compositional stability while eliminating secondary phase risks. The methodology establishes a universal framework for defect engineering in chalcogenide photovoltaics, bridging fundamental insights into alkali metal diffusion thermodynamics with scalable manufacturing solutions. These findings advance kesterite solar cell technology and offer a blueprint for optimizing thin‐film devices, improving process tolerance and material sustainability.

## Graphical abstract

This study contrasts conventional processing with rapid‐cooling pathways for CZTSSe absorber fabrication. Visualized through dual synthesis routes, the Na‐locking strategy utilizes accelerated cooling rates to regulate Na diffusion from soda‐lime glass. Enhanced grain growth, reduced defects, and a denser absorber layer yield a 13.22%‐efficiency solar cell, with improved voltage and fill factor. The gentle approach minimizes structural disruption, offering a practical route for high‐performance thin‐film photovoltaics.

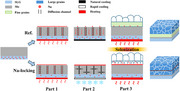

## 1 Introduction

Given the higher abundance of Zn and Sn in the Earth's crust compared to the much scarcer In and Ga in Cu(In,Ga)Se_2_, Cu_2_ZnSn(S,Se)_4_ (CZTSSe) emerges as a more cost‐effective and suitable material for large‐scale production of thin‐film solar cells.^[^ ^1^, ^2^, ^3^, ^4^, ^5^ ^]^ By adjusting the ratio of S to Se, the bandgap of CZTSSe can be finely tuned between 1.0 and 1.5 eV, achieving optimal matching for the absorption of different wavelengths of sunlight across the solar spectrum.^[^ ^6^, ^7^, ^8^, ^9^, ^10^ ^]^ In theory, the power conversion efficiency (*PCE*) of CZTSSe cells can reach 32%,^[^ ^11^, ^12^ ^]^ while the highest efficiency achieved in laboratory settings is only 15.1%, resulting in a significant gap between theory and practice.^[^ ^13^ ^]^ It is widely recognized that the primary bottlenecks are the low open‐circuit voltage (*V_OC_*) and insufficient fill factor (*FF*). To address these issues, researchers have developed various strategies, including cation substitution (e.g., doping with Ag, Cd, Ge, etc.) and interface engineering, among others.^[^ ^14^, ^15^, ^16^, ^17^, ^18^, ^19^, ^20^ ^]^

The application of Ag doping technology in CZTSSe solar cells has become highly mature, with its effectiveness in passivating deep‐level defects and improving material performance being extensively validated by numerous studies.^[^ ^21^, ^22^, ^23^, ^24^, ^25^, ^26^, ^27^, ^28^ ^]^ By appropriately adjusting the doping concentration and distribution of Ag, significant enhancements in the open‐circuit voltage and conversion efficiency of the solar cells can be achieved. In view of the widespread applicability and notable advantages of this technology, the present study employs Ag doping alongside additional optimizations, particularly Na incorporation, to achieve more efficient defect passivation and performance improvement.

In the published literature, referring to the introduction of Na into CZTSSe as “Na doping” might not be entirely accurate. This is because, in practice, Na does not actually enter the lattice structure of CZTSSe—it neither replaces the original atomic units to occupy corresponding lattice sites nor takes up interstitial positions within the lattice. Instead, Na is more likely to accumulate at grain boundaries (GBs).^[^ ^29^, ^30^, ^31^, ^32^, ^33^, ^34^ ^]^ This phenomenon also explains well why no shift in XRD peak positions has been observed after introducing Na into the CZTSSe absorber. As is well known, Na can provide one electron (thereby adopting a +1 oxidation state) to combine with dangling bonds. Through this bonding method, it effectively passivates deep‐level defects at GBs, thereby increasing carrier lifetime. This is also a major reason why most CZTSSe solar cells use soda‐lime glass as the substrate.^[^ ^35^, ^36^, ^37^ ^]^ Specifically, when using soda‐lime glass as the substrate, under high‐temperature conditions, Na diffuses toward the absorber layer. This diffusion behavior promotes the growth of CZTSSe grains and passivates interface defects. However, in previous studies, researchers have often merely used heating methods to promote such Na diffusion without paying attention to actively controlling the cooling rate, thus failing to effectively regulate this diffusion process.^[^ ^38^ ^]^In fact, Na diffusion holds great potential for improving cell performance, but this potential has yet to be fully explored.

This study aims to investigate the impact of “Na locking” technology on the performance of CZTSSe thin‐film solar cells. Building upon prior work involving Ag doping, we adopted a strategy that combines rapid and slow cooling to lock Na atoms at the surface of the Mo back electrode, preventing their backward diffusion and thus achieving a Na locking effect. This strategy would lead to the accumulation of Na in the bottom region of the CZTSSe layer, thereby promoting grain growth in that area. Additionally, during the spin‐coating process, extended sintering was performed on each precursor layer to promote the further diffusion of Na atoms deeper into the absorber, which not only increased the grain size at the top layer but also provided a higher‐quality deposition substrate for the CdS buffer layer.^[^ ^39^, ^40^ ^]^ By optimizing the aforementioned processes, a substantial increase in the grain size across the entire absorber was achieved. This enhancement effectively minimized non‐radiative recombination within the material and improved carrier transport efficiency. Ultimately, these improvements collectively contributed to the best *PCE* of 13.22% for the solar cell device (without the MgF_2_ anti‐reflection layer) prepared in this study. The findings underscore the critical role of Na locking technology in boosting the performance of CZTSSe thin‐film solar cells.

## 2 Results and Discussion

### 2.1 Control Mechanism of Na Diffusion in Mo Sheets and Its Impact on Precursor Film Characteristics

**Figure 1:**
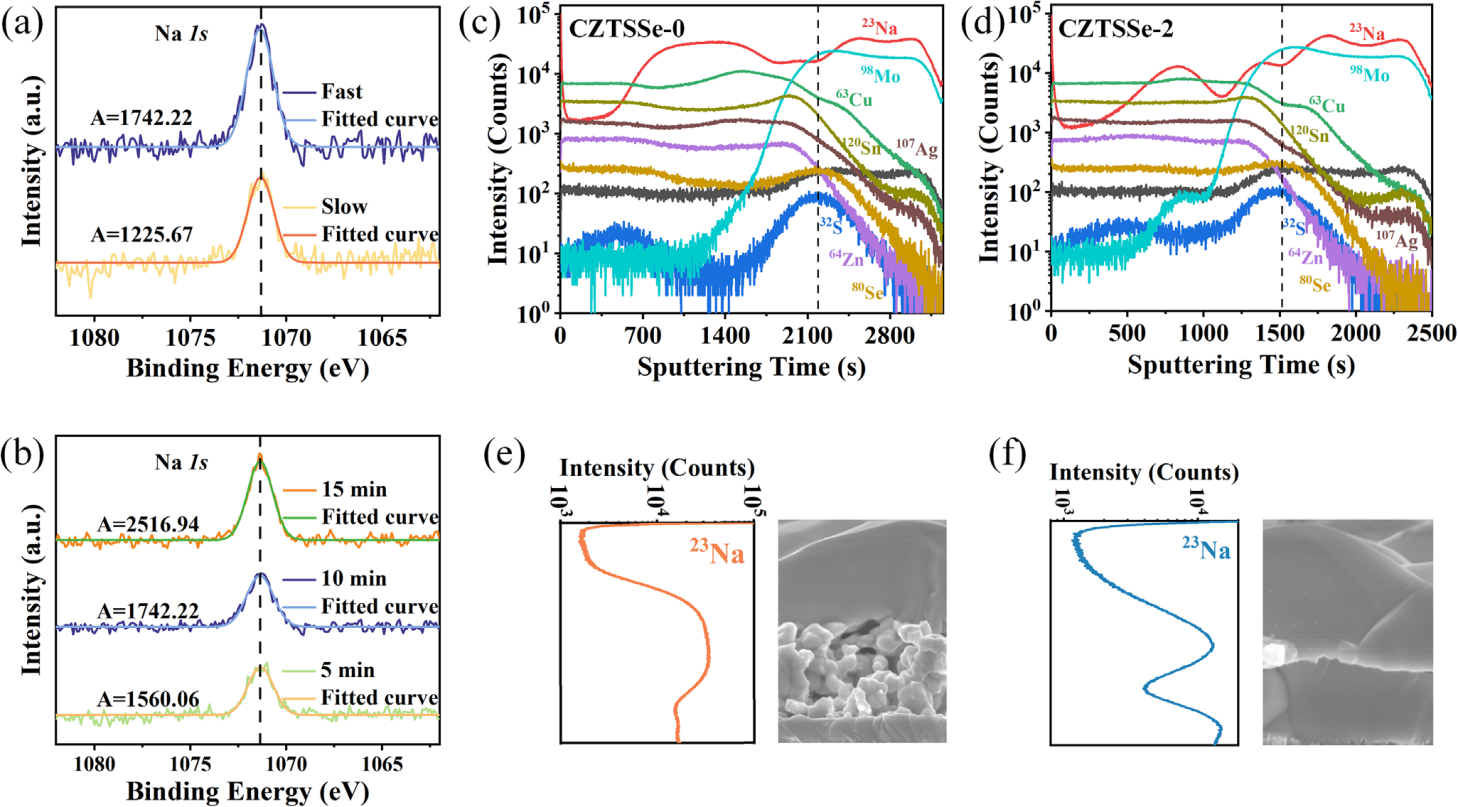
XPS analysis of the Na *1s* signal in the Mo surface layer under a) different heating times and b) varying cooling rates. The labeled A values correspond to the integrated peak areas. TOF‐SIMS depth profiles of c, e) CZTSSe‐0 and d, f) CZTSSe‐2. The cross‐sectional morphology images placed alongside are used to assist in determining the depth position of the elemental distribution.

During our experimental process, it was observed that under slow cooling conditions, some Na would re‐enter the glass substrate as the temperature decreased. To investigate the impact of cooling rate on the final Na content in the samples, a series of comparative experiments using both rapid cooling and natural cooling methods were designed and conducted. The Mo sheets with different cooling rates were labeled as “Fast” (rapidly cooled) and “Slow” (naturally cooled), respectively. XPS analysis revealed a higher Na content in the Mo sheet subjected to rapid cooling compared to that in the naturally cooled samples, as shown in **Figure** **1**a. To further confirm the XPS results, energy‐dispersive X‐ray spectroscopy (EDS) analysis was also performed. The EDS measurements consistently revealed higher Na content in the rapidly cooled Mo sheets compared to the naturally cooled ones, as shown in Figure S1a,b (Supporting Information), aligning well with the XPS observations. The rise in Na content within Mo suggests that more Na will migrate into the absorber layer during selenization. This indicates that rapid cooling immediately after sintering can effectively accelerate the closure of diffusion channels, thereby retaining more Na within the absorption layer and achieving the goal of Na locking. The corresponding solar cell efficiency statistics in Figure S1c (Supporting Information) confirm the effectiveness of this approach in enhancing device performance. Rapid cooling during precursor preparation does not induce defects in the final selenized absorber, as evidenced by admittance spectroscopy (Figure S2, Supporting Information).

To investigate and control the extent of Na diffusion, the sintering time was extended, and the Na content in Mo was measured using XPS, as illustrated in Figure 1b. Sintered Mo sheets were designated as *x* min (*x* = 5, 10, 15), where *x* denotes the sintering time in minutes, at 300 °C. Clearly, as the sintering time increases, more Na diffuses to the surface of Mo. At a sintering time of 15 min, there is significant Na enrichment in the Mo surface layer. Notably, the extension of sintering time promotes Na diffusion, and within the experimental time range (up to 15 min), no limit to Na diffusion was observed. In addition, we further analyzed the Na content through EDS measurements, which confirmed consistent results with the XPS analysis, demonstrating a time‐dependent Na accumulation trend in the Mo surface layer. Detailed EDS characterization data can be found in Figure S1 and Table S1 (Supporting Information).

The XPS spectrum of the Na *1s* core level exhibits a binding energy of 1071.5 eV, which unambiguously corresponds to metallic Na. According to bond energy data from the CRC Handbook, the Mo‐O bond (≈502 kJ mol^−1^) exhibits substantially higher strength compared to the Na‐O bond (≈270 kJ mol^−1^). Notably, Na_2_O is one of the primary constituents in soda‐lime glass substrates. This energy disparity creates a thermodynamic driving force for oxygen atom migration from Na_2_O to Mo under high‐temperature conditions, forming metallic Na and MoO*_x_* compounds through an ionic exchange mechanism. During this process, elemental Na is continuously released from the soda‐lime glass substrate and transported to the surface via diffusion channels within the Mo matrix. Unlike conventional methods involving the direct addition of Na^+^‐containing inorganic salts, the in situ generated Na exists in a non‐free state protected by surrounding Mo atoms. This unique coordination environment effectively prevents parasitic reactions with atmospheric O_2_ and H_2_O while preserving Na's capacity to donate valence electrons ‐ a critical feature that ensures optimal sodium passivation performance in subsequent absorber layers.

During the preparation of CZTS precursors on Mo‐coated substrates, there is a high‐temperature (300 °C) drying step to remove the organic solvent. This sintering process clearly promotes Na diffusion toward the top of the CZTS precursor. However, at 300 °C, Na fails to migrate to the precursor surface, as evidenced by the absence of Na 1s signal in the XPS analysis of the obtained CZTS thin film (Figure S3, Supporting Information). As previously discussed, rapid cooling combined with extended sintering times can promote more Na from the substrate through the Mo layer to the surface, thereby increasing the Na content in the CZTS precursor. Figure S4 (Supporting Information) shows the surface and cross‐sectional morphologies of CZTS precursors prepared with rapid cooling and different sintering times. It can be observed that the surface roughness is slightly mitigated, as shown in Figures S5 and S6 (Supporting Information).

### 2.2 Influence of Na Diffusion on Properties of CZTSSe Absorber Layer

**Figure 2:**
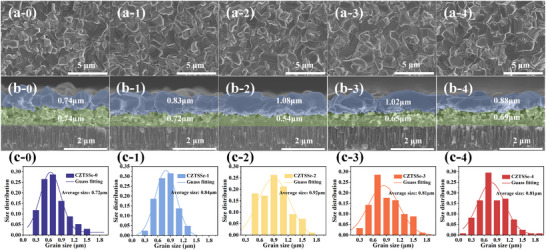
Surface a) and cross‐sectional b) morphology and average grain size c) of CZTSSe with different extended sintering times.

**Figure 3:**
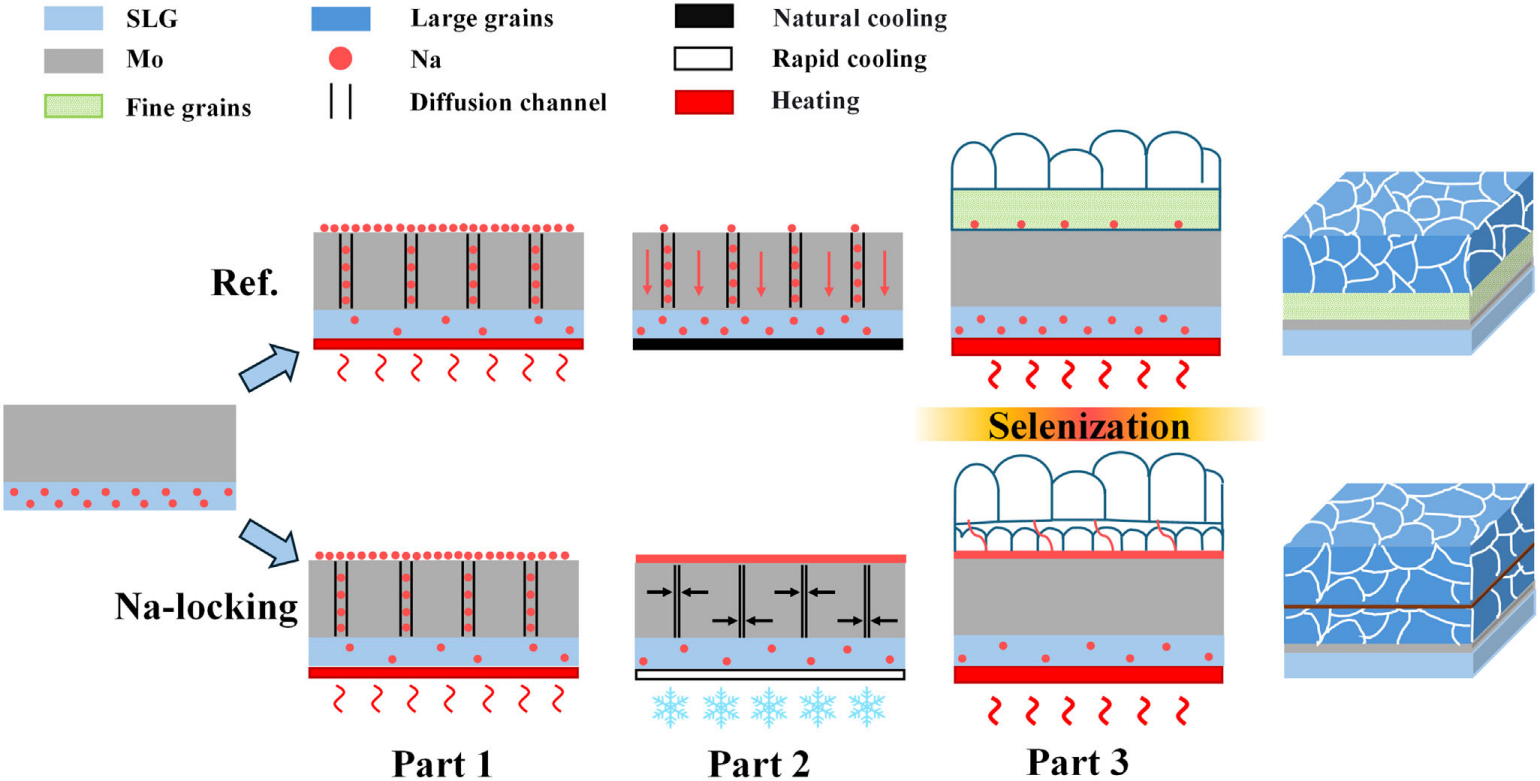
Schematic comparison of grain growth mechanisms in CZTSSe prepared by Na‐locking strategy and conventional methods.

As shown in the surface morphology in **Figure** **2**a, all films exhibit a dense structure without visible pinholes. In the previous section, it has been demonstrated that prolonged sintering time enhances upward‐directed Na diffusion. Figure 2b illustrates that increased Na diffusion into the absorber layer promotes pronounced lateral growth of surface grains, resulting in a significant increase in average grain size, as statistically confirmed in Figure 2c. Furthermore, variations in Na content substantially alter the microstructure in the bottom region of CZTSSe absorbers. Notably, higher Na content does not always yield better performance; instead, an optimal Na concentration exists. Appropriate Na content facilitates the formation of a relatively dense bilayer structure within the absorber, characterized by fewer voids at internal interfaces. In this study, CZTSSe‐2 exhibits the optimal bilayer dense morphology with minimal interlayer voids, corresponding to the solar cell device with the highest efficiency. The grain growth mechanism of CZTSSe under this Na‐locking strategy is depicted in **Figure** **3**.

SIMS analysis (Figure 1c–f) combined with SEM cross‐sectional morphology (Figure 2) reveals significantly higher Na concentrations in fine‐grained regions of the absorber layer compared to large‐grained regions. This is attributed to the higher grain boundary density in fine‐grained regions, which provides more accommodation sites for Na. Although Na may diffuse toward the surface and GBs during the precursor stage, the growth and densification of upper‐layer grains during selenization reduce grain boundary density, compressing Na's available space and driving its migration toward the absorber layer bottom. This explains the preferential growth of small grains in Na‐rich bottom regions.

**Figure 4:**
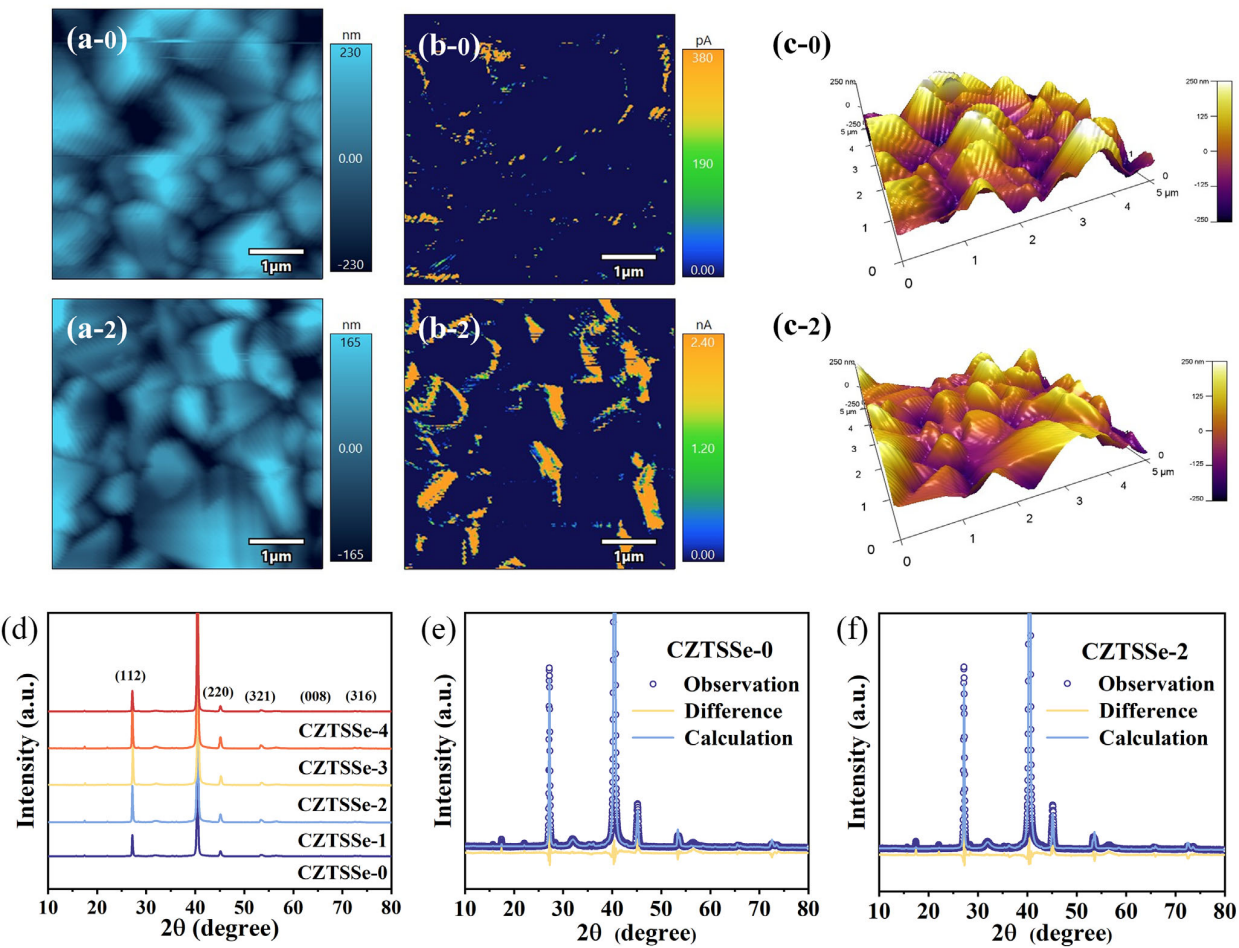
a) Surface roughness, b) surface current distribution, and c) 3D profiles of CZTSSe precursors with different extended sintering times. d) XRD patterns of CZTSSe with different extended sintering times; e, f) Refinement of XRD patterns for CZTSSe films with different extended sintering times.

**Figures** **4**a–c and S7 (Supporting Information) present the surface roughness, average surface current distribution, and 3D profiles of CZTSSe films, offering an intuitive visualization of surface morphology. Samples with different sintering times reveal that CZTSSe‐2 exhibits significantly enhanced surface conductivity compared to others. The outstanding device performance originates from the compact grain arrangement in the underlying layer and minimized interlayer voids, which synergistically enhance carrier transport dynamics. This optimized microstructure in CZTSSe‐2 not only improves charge carrier collection efficiency but also extends carrier lifetimes, thereby accounting for the reduced reverse saturation current density (*J_0_*) observed in subsequent experiments. Additionally, Figure S8 (Supporting Information) provides statistical analysis of average roughness and surface current values in observed regions, further confirming that CZTSSe‐2 indeed has the lowest roughness and highest average surface current.

The XRD results (Figure 4c) reveal that all diffraction peaks correspond exclusively to CZTSSe and Mo, with no secondary phases detected in the CZTSSe films, confirming their high material purity. Rietveld refinement of the XRD data, as shown in Figure 4d,e and Figure S9 (Supporting Information), demonstrates good agreement between the structural model and experimental data, supported by the *R_exp_* (expected reliability factor), *R_wp_* (weighted profile factor), *R_p_* (profile factor), and *GOF* (goodness‐of‐fit) values. The refined grain size indicates that the CZTSSe‐2 sample exhibits optimal crystallinity. Notably, a discrepancy exists between the grain sizes calculated from XRD and those measured by SEM. This difference arises because SEM quantifies the lateral dimensions of grains, whereas XRD reflects their longitudinal size. Specific values are provided in **Table** **1** and Table S2 (Supporting Information). Larger grain sizes generally correlate with fewer GBs, reducing scattering centers and defects, which enhances material electrical properties and facilitates efficient charge transport.

**Table 1:** Refinement results of XRD data for CZTSSe with different extended sintering times.

Sample	Grain size [nm]	*R_exp_*	*R_wp_*	*R_p_*	*GOF*
CZTSSe‐0	41.18	2.93	11.32	6.63	3.86
CZTSSe‐2	42.75	2.93	12.09	7.02	4.12

Additionally, the lattice constants of the CZTSSe film remain approximately *a* = *b* = 5.70 Å and c = 11.35 Å, showing no detectable variation. The absence of peak shift in the CZTSSe (112) diffraction pattern suggests that Na does not substitute into the host lattice. These structural observations strongly suggest that Na preferentially segregates at GBs rather than participating in lattice formation.

### 2.3 Effect of Na Diffusion on the Photovoltaic Performance of CZTSSe Solar Cells

**Figure 5:**
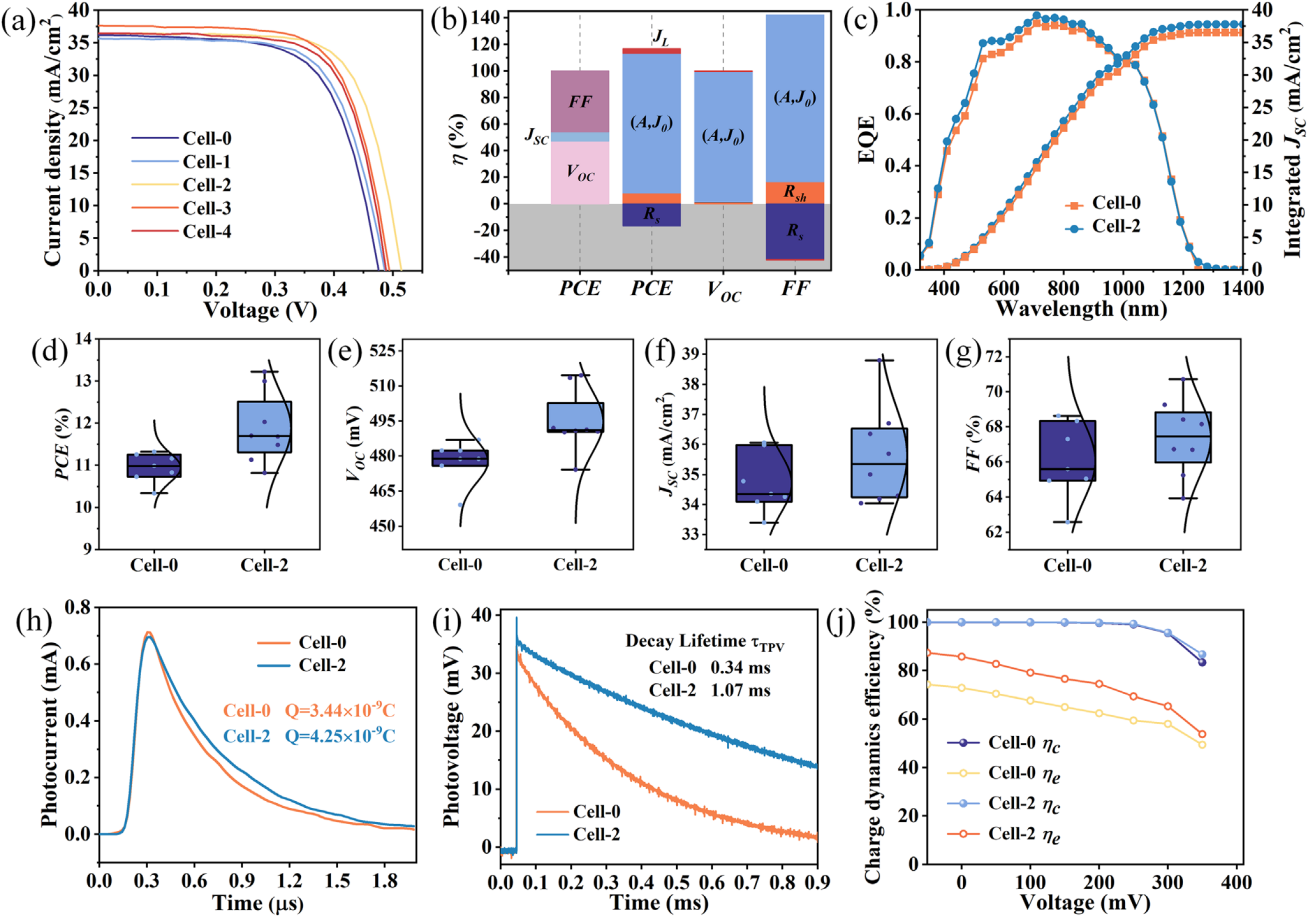
a) *J–V* curves of CZTSSe solar cells with extended sintering times (0–4 min, labeled Cell‐0 to Cell‐4) under AM1.5G illumination. b) Relative contributions of photovoltaic parameters to device performance for Cell‐0 and Cell‐2. c) EQE spectra and integrated *J_SC_* for Cell‐0 and Cell‐2. d–g) Statistical distributions of *PCE*, *V_OC_*, *J_SC_*, and *FF*. h) TPC and i) TPV decay curves, along with j) the corresponding *η_c_* and *η_e_* parameters derived from these measurements for Cell‐0 and Cell‐2 devices.

**Figure** **5**a,d–g illustrate the variations in photovoltaic performance parameters of CZTSSe devices under different extended sintering times, including *V_OC_*, short‐circuit current density (*J_SC_*), fill factor (*FF*), and *PCE*. **Table** **2** summarizes the statistical results of photovoltaic parameters for five devices (Cell‐0, Cell−1, Cell‐2, Cell‐3, and Cell‐4). Notably, the *PCE* of device Cell‐2 significantly improved from 11.30% to 13.22%, with *V_OC_* increasing from 475.94 to 513.9 mV, *J_SC_* rising from 36.15 to 36.37 mA·cm^−2^, and *FF* growing from 65.7% to 70.70%. As shown in Figure 5c, Cell‐2 exhibits markedly enhanced EQE in the visible spectrum compared to Cell‐0. The high absorption coefficient in this region enables photon absorption primarily within the absorber surface layer, where the generated photocarriers are more efficiently collected by electrodes. This improved carrier collection efficiency directly reflects suppressed carrier recombination and enhanced charge separation/transport processes, likely attributable to Na passivation at GBs. According to the research literature published by Yao's group,^[^ ^42^ ^]^ the contribution rates of corresponding parameters to the performance improvement were calculated and displayed in Figure 5b. It is evident that the improvement in *PCE* of solar cells can be attributed to the synergistic enhancement of *V_OC_* and *FF*, which fundamentally reflects the optimization of recombination parameter *A* and *J_0_*. According to the single‐junction cell physical model, *J_0_* is determined by both exponential and pre‐exponential factor:

(1) $$J_{0}=J_{00}e^{-\frac{E_{g}}{AkT}}$$

where the pre‐exponential factor *J_00_* is given by:

(2) $$J_{00}=qN_{C}N_{V}\left[\frac{1}{N_{A}}\sqrt{\frac{D_{n}}{\tau_{n}}}+\frac{1}{N_{D}}\sqrt{\frac{D_{p}}{\tau_{p}}}\right]$$

here, *N_C_* and *N_V_* represent the effective density of states in conduction/valence bands, *N_A_*/*N_D_* denotes acceptor/donor concentrations, and *D_n_*/*D_p_*, *τ_n_*/*τ_p_* correspond to carrier diffusion coefficients and lifetimes.

**Table 2:** Detailed photovoltaic parameters of CZTSSe solar cells with different extended sintering.

Device	*V_OC_* [mV]	*J_SC_* [mA cm^−2^]	*FF* [%]	*PCE* [%]	*R_S_* [Ω·cm^2^]	*R_Sh_* [Ω·cm^2^]	*A*	*J_0_* [mA/cm^2^]
Cell‐0	475.94	36.15	65.70	11.30	0.74	874.44	1.72	7.58×10^−5^
Cell‐1	486.30	35.65	67.60	11.72	1.16	1952.45	1.44	7.20×10^−5^
Cell‐2	513.90	36.37	70.70	13.22	1.02	9153.48	1.41	2.27×10^−5^
Cell‐3	494.14	37.55	68.60	12.72	1.16	1952.45	1.49	9.05×10^−5^
Cell‐4	488.01	36.32	69.40	12.30	0.81	2002.60	1.47	8.25×10^−4^

Equations (1) and (2) reveal a strongly coupled relationship between parameters A and *J_0_*, rather than their independence. This inherent interdependence necessitates comprehensive joint evaluation when assessing their contributions to device performance. Theoretical analysis reveals that the pre‐exponential factor *J_00_* is critically dependent on carrier lifetimes (*τ_n_*, *τ_p_*) and defect‐mediated recombination. The Na diffusion‐based grain boundary passivation process effectively suppresses Shockley‐Read‐Hall (SRH) recombination, as evidenced by the continuous reduction of A values. The reduced defect density significantly prolongs carrier lifetimes, directly lowering *J_00_* through Equation (2), while the suppressed recombination mechanisms simultaneously reduce parameter A, which in turn exponentially decreases *J_0_* according to the dependence expressed in Equation (1). This optimization of recombination dynamics ultimately leads to simultaneous enhancement of *V_OC_* and *FF*, which shows mechanistic consistency with the experimentally observed reductions in *V_OC_* and *FF* losses. To isolate and verify the actual impact of Na on solar cell performance and clarify whether synergistic doping of Na and Ag can further enhance device efficiency, control solar cells were fabricated using quartz substrates without intrinsic Na under identical preparation conditions. Experimental results demonstrated that the *PCE* of solar cells prepared with quartz substrates decreased significantly by ≈33% (Figure S10 and Table S3, Supporting Information), indicating that Na incorporation plays a crucial role in enhancing photovoltaic performance.

M‐TPC (Modulated Transient Photocurrent) and TPV (Transient Photovoltage) techniques serve as critical tools for characterizing charge transport dynamics and recombination losses in solar cells. Specifically, M‐TPC evaluates device performance by monitoring the short‐circuit current response under pulsed light excitation. Through integration of the current decay curve over time, the total charge (Q) extracted at the device terminals can be quantitatively determined. As shown in Figure 5h, Cell‐2 exhibits a significantly higher accumulated charge of 4.25 nC compared to Cell‐0's 3.44 nC, revealing its superior capability in photogenerated carrier capture and collection. This enhancement originates from the effective suppression of interfacial recombination in Cell‐2, which aligns with the conclusions discussed in the preceding section. Correspondingly, M‐TPV characterizes the voltage response of solar cells under pulsed illumination in an open‐circuit configuration. Since the external circuit is open, the photogenerated charges collected at both electrodes after pulsed light illumination can only be consumed through recombination processes within the cell. Consequently, a longer TPV lifetime (*τ_TPV_*) derived from photovoltage decay kinetics indicates a slower recombination rate of photogenerated charges and diminished carrier loss during device operation. Figure 5i shows that after introducing Na, the τ_TPV_ increased from 0.34 ms to 1.07 ms, evidencing substantial suppression of non‐radiative recombination pathways during charge transport.

Based on the M‐TPC/TPV curves, the extraction efficiency (*η_e_*) and collection efficiency (*η_c_*) of the devices were further calculated, with results shown in Figure 5j. It is evident that the extraction efficiency of Cell‐0 is significantly lower than that of Cell‐2, reaffirming the notable advantage of Cell‐2 in extracting photogenerated carriers. The enhancement in Cell‐2's ability to extract and collect photogenerated carriers is closely related to the suppression of interface recombination mentioned earlier, which is also the primary reason for its improved *V_OC_* and *FF*. Figure S11 (Supporting Information) presents the results of EIS, with the equivalent circuit diagram showing *C* as the junction capacitance, *R_ct_* as the recombination resistance, and *R_0_* as the series resistance. According to the formula *τ* = *C* × *R_ct_*, when the device's *R_ct_* increases, the minority carrier lifetime correspondingly increases, consistent with the previously mentioned increase in *τ_TPV_*, further confirming that carrier recombination has indeed been suppressed.

**Figure 6:**
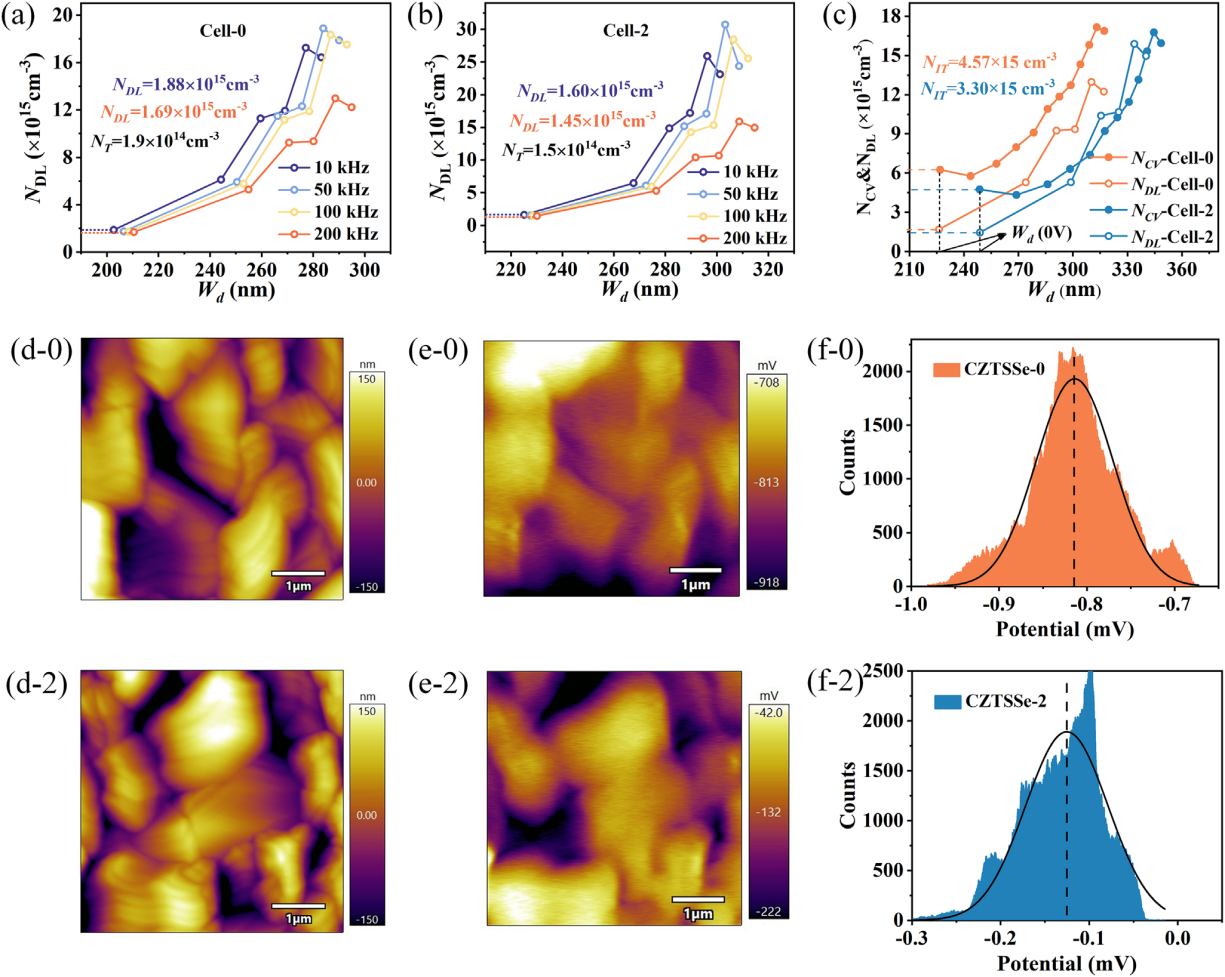
DLCP profiles measured at frequencies ranging from 10 to 200 kHz for a) Cell‐0 and b) Cell‐2 devices; c) Comparison of *N_CV_* (from C‐V) and *N_DL_* (from DLCP) for both devices; d) Topography images; e) surface potential maps, and f) the mean contact potential differences (CPD) of CZTSSe‐0 and CZTSSe‐2, calculated by averaging the values at each pixel in each film.

C‐V and DLCP are used to characterize the depletion layer width, carrier concentration, interface defect density, and bulk defect density of the devices.^[^ ^41^ ^]^ Specific data is shown in **Table** **3**. **Figure** **6**a,b show that after the introduction of Na, the defect density *N_DL_* (at 200 kHz) gradually decreases from 1.69 × 10^15^ to 1.45 × 10^15^ cm^−3^, and the bulk defect concentration *N_T_* (the difference between *N_DL_* at 10 kHz bias and 200 kHz bias) gradually decreases from 1.90 × 10^14^ to 1.50 × 10^14^ cm^−3^. According to the data in Figure 6c, the difference between *N_C‐V_* and *N_DL_* is the interface defect concentration (*N_IT_*), which decreases from 4.57 × 10^15^ cm^−3^ to 3.30 × 10^15^ cm^−3^. This reduction further corroborates the decreasing trend of the ideality factor A<2 discussed earlier.

**Table 3:** C‐V and DLCP derived electrical parameters for Cell‐0 and Cell‐2 devices.

Device	*N_CV_* [cm^−3^]	*N_DL_* [cm^−3^]	*N_IT_* [cm^−3^]	*N_T_* [cm^−3^]	*W_d_* [nm]
Cell‐0	6.26×10^15^	1.69×10^15^	4.57×10^15^	1.90×10^14^	227
Cell‐2	4.75×10^15^	1.45×10^15^	3.30×10^15^	1.50×10^14^	249

By comparing Figure 6d–f, it is evident that the potential at GBs is significantly lower than in grain interiors, indicating the presence of negative charge centers at these boundaries. These negative charges likely originate from dangling bonds or defects at GBs, which can hinder hole transport and enhance carrier recombination probability. Notably, the grain boundary potential of CZTSSe‐2 is substantially higher than that of CZTSSe‐0, explaining why CZTSSe‐2 exhibits an overall higher potential distribution. This phenomenon suggests that increased Na content significantly passivates grain boundary defects. As a typical polycrystalline material, CZTSSe contains abundant unsaturated dangling bonds at GBs and surfaces. These structural defects create pronounced hole carrier potential wells in boundary and surface regions. Na can neutralize unpaired electrons in dangling bonds via direct electron donation, reducing charge trap state density at these regions and ultimately elevating their electrostatic potential (i.e., achieving grain boundary passivation). A higher contact potential difference (CPD) indicates a higher Fermi level and a lower work function. Clearly, the CZTSSe‐2 absorber layer exhibits a higher surface Fermi level, suggesting a lower carrier concentration (for p‐type material), which can result in a wider depletion layer width when forming a p‐n junction, beneficial for the separation of photogenerated carriers. This observation is consistent with the aforementioned C‐V and DLCP results.

## 3 Conclusion

This study demonstrates that strategic control of Na diffusion dynamics is essential for unlocking the full potential of CZTSSe photovoltaic devices. By synergistically optimizing sintering duration and cooling protocols, we establish a thermally activated pathway that governs Na migration from the soda‐lime glass substrate into the absorber layer. The rapid cooling mechanism effectively terminates back‐diffusion processes, anchoring Na at critical interfacial regions where it serves dual functions: passivating electronically active defects at grain boundaries and modulating crystallization kinetics during selenization. This approach not only enhances morphological homogeneity through promoted lateral grain growth but also suppresses carrier recombination by restructuring the energetic landscape at grain interfaces. The resultant absorber layer exhibits improved crystallographic integrity and interfacial charge transport characteristics, directly correlating with the observed *V_OC_* and *FF* enhancements. Notably, the efficiency breakthrough to 13.22% underscores the importance of spatially controlled Na distribution rather than absolute concentration. These findings redefine the role of alkali metal management in kesterite photovoltaics, emphasizing defect engineering through diffusion pathway manipulation as a critical design principle. The developed methodology provides a universal framework for interfacial optimization in solution‐processed thin‐film solar cells, bridging fundamental insights into defect chemistry with practical device engineering strategies.

## 4 Experimental Section

### Preparation of (Cu,Ag)_2_ZnSnS_4_ Precursor Films

To prepare the precursor solution, 0.6585 g of ZnCl_2_, 0.8940 g of SnCl_4_·2H_2_O, 0.3593 g of CuCl, and 1.5186 g of thiourea were directly added to 10 mL of 2‐methoxyethanol. After stirring for 0.5 h at 60 °C, 0.0579 g of AgCl was added and continued stirring for another 1 h, thereby successfully preparing the Ag‐containing Cu_2_ZnSnS_4_ precursor solution. Before spin‐coating, the cleaned Mo‐coated substrates were preheated for 10 min to increase the Na content in the Mo surface layer(the absence of a SiO_2_ barrier layer in the Mo layer does not affect the upward diffusion of Na from the soda‐lime glass). Subsequently, a Na‐free precursor solution was spin‐coated onto the Mo‐coated soda‐lime glass substrates and heated at 300 °C for 2 min to form the precursor film; the film prepared under these conditions served as the reference sample and was named Pre‐0. Similarly, samples prepared with extended sintering times of 1, 2, 3, and 4 min were named Pre‐*x* (*x* = 1, 2, 3, 4). All steps were carried out in ambient air conditions.

### Device Fabrication

The precursor film underwent high‐temperature selenization in a Se‐containing graphite box at 550 °C for 900 s, forming an Ag‐doped CZTSSe absorber layer. Since the amount of Ag source was consistent across all absorber layer preparation conditions, the Ag‐doping will not be further emphasized in the following text. The CdS buffer layer was deposited on the CZTSSe using a chemical bath deposition method with a mixed solution of CdSO_4_, thiourea, and NH_4_OH. Subsequently, the i‐ZnO and ITO layers were prepared by magnetron sputtering, and the Ag electrode was deposited by evaporation. This process ultimately yielded CZTSSe devices with structures of SLG/Mo/CZTSSe/CdS/i‐ZnO/ITO/Ag. The active area of the CZTSSe solar cell was 0.19 cm^2^.

### Characterizations

Structural characterization was conducted using X‐ray diffraction (XRD) with a Bruker D8 Advance diffractometer, utilizing Cu K_α_ radiation (λ = 1.5406 Å) over a 2θ range of 10° to 80° with a step size of 0.02°, to ensure precise material analysis. The surface morphology and cross‐sectional views were observed via scanning electron microscopy (SEM) using a Zeiss Merlin Gemini II. Surface roughness, electrical current, and surface potential were measured using atomic force microscopy (AFM), conductive AFM (c‐AFM), and Kelvin probe force microscopy (KPFM) on a Cypher S AFM (Asylum Research, Oxford Instruments). The external quantum efficiency (EQE) was measured with a Newport Quant X‐300. The current–voltage (*I–V*) characteristics of the device performance were assessed under standard AM 1.5G illumination (100 mW·cm^−2^). Electrochemical impedance spectroscopy (EIS) was conducted in dark conditions with a 0V bias.

## Conflict of Interest

The authors declare no conflict of interest.

## Supporting information



Supporting Information

## Data Availability

Research data are not shared.
