# Supplementary material for: Unveiling Sodium Diffusion Kinetics and Locking Mechanisms for High‐Performance CZTSSe Photovoltaics
Source: Adv Sci (Weinh). 2025 May 23;12(30):e04087. doi: 10.1002/advs.202504087 (PMC12376674; doi:10.1002/advs.202504087)
Supplement: Supplementary file 1 — Supporting Information [file ADVS-12-e04087-s001.docx]

Supporting Information

**Unveiling Sodium Diffusion Kinetics and Locking Mechanisms for High-Performance CZTSSe Photovoltaics**

*Shuyu Li, Chaoran Li, Chu Liu, Jiachen Wu, Letu Siqin, Yuan Li, Guonan Cui, Yanchun Yang^,^, Ruijian Liu^*^, Hongmei Luan, Chengjun Zhu**

Shuyu Li, Chaoran Li, Chu Liu, Jiachen Wu, Letu Siqin, Yuan Li, Ruijian Liu*, Hongmei Luan, Chengjun Zhu*

Inner Mongolia Key Laboratory of Semiconductor Photovoltaic Technology and Energy Materials, Center for Quantum Physics and Technologie, School of Physical Science and Technology, Inner Mongolia University, 010021, China

E-mail: [cjzhu@imu.edu.cn](mailto:cjzhu@imu.edu.cn); ruijian-liu@imu.edu.cn

Guonan Cui, Yanchun Yang

School of Physics and Electronic Information, Inner Mongolia Normal University, Inner Mongolia 010022, China


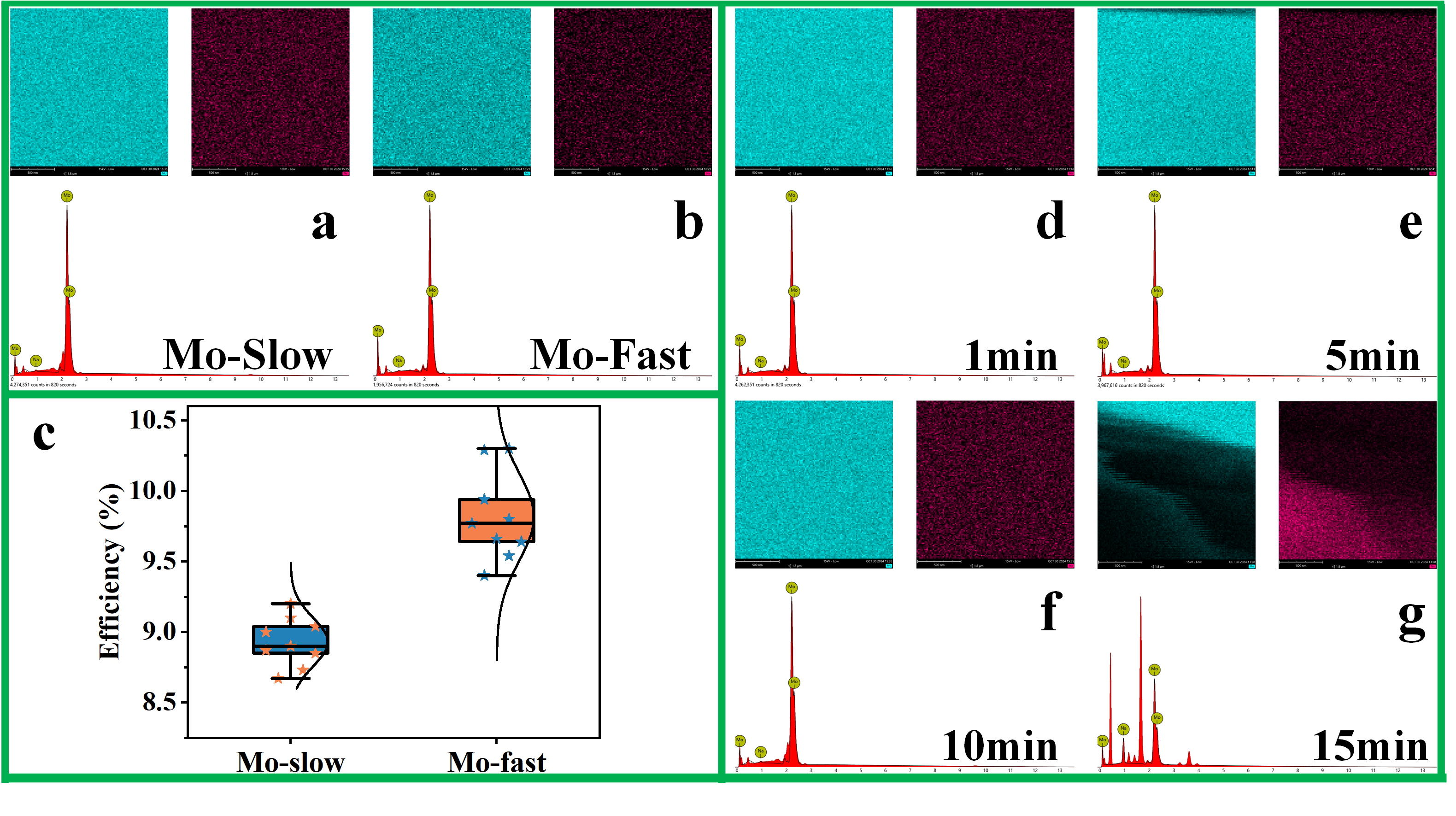


**Figure S1.** (a,b) EDS analysis of Na distribution in Mo sheets with different cooling rates; (c) *PCE* statistics for solar cells with different Mo substrates; (d-g) Elemental distribution maps of Mo and Na in Mo sheets with different sintering times.

**Table S1.** Na content in Mo sheets with different cooling rates measured by EDS.

| Sample | Mo  (at.%) | Na  (at.%) |
| --- | --- | --- |
| Mo-slow | 98.99 | 1.01 |
| Mo-fast | 98.10 | 1.90 |
| Mo-1 | 99.14 | 0.86 |
| Mo-5 | 98.98 | 1.02 |
| Mo-10 | 98.10 | 1.90 |
| Mo-15 | 67.16 | 32.84 |


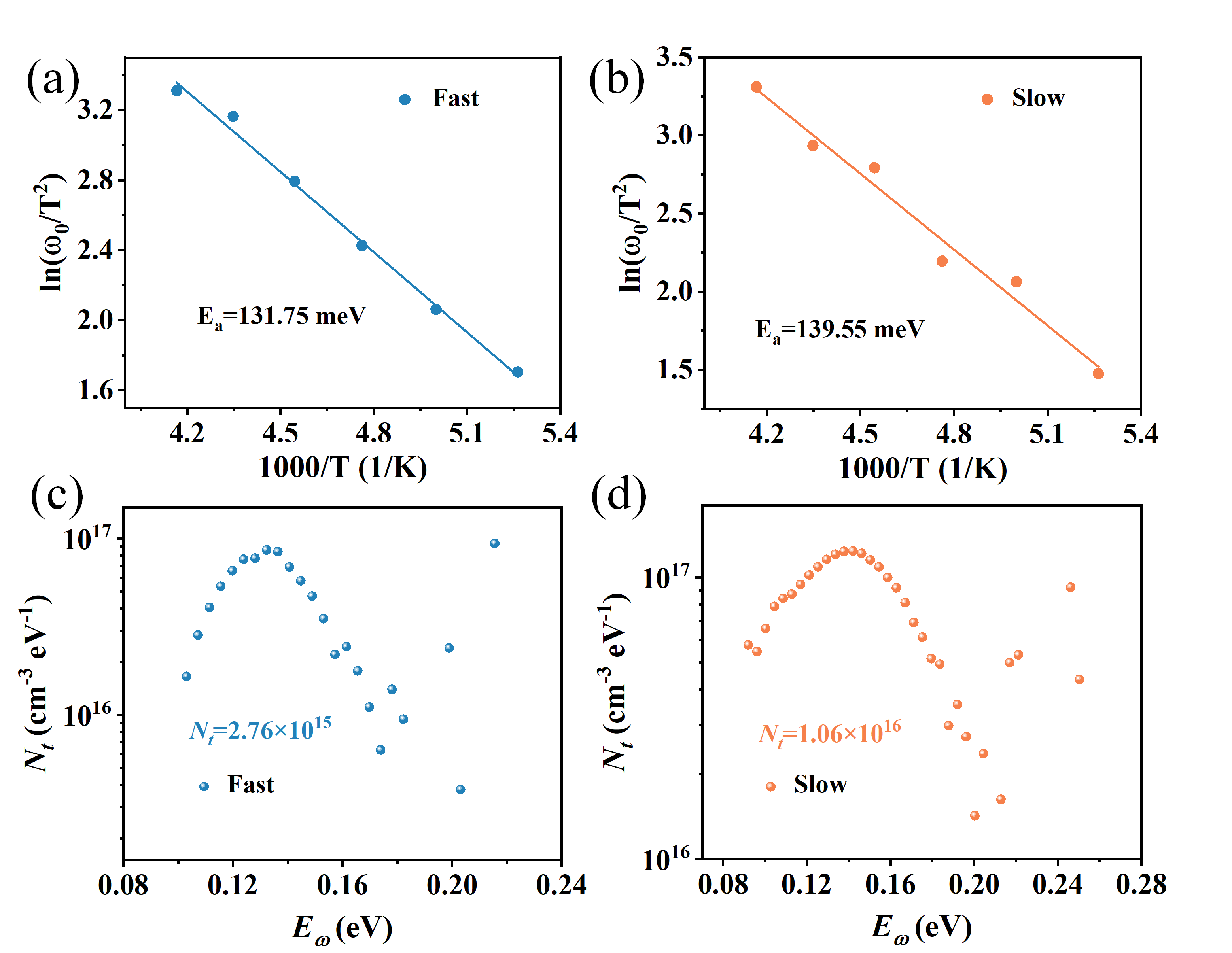


**Figure S2.** Arrhenius plots of the inflection point frequencies determined from the admittance results of (a) Fast and (b) Slow devices. Defect density spectra derived from the admittance spectra of (c) Fast and (d) Slow devices.


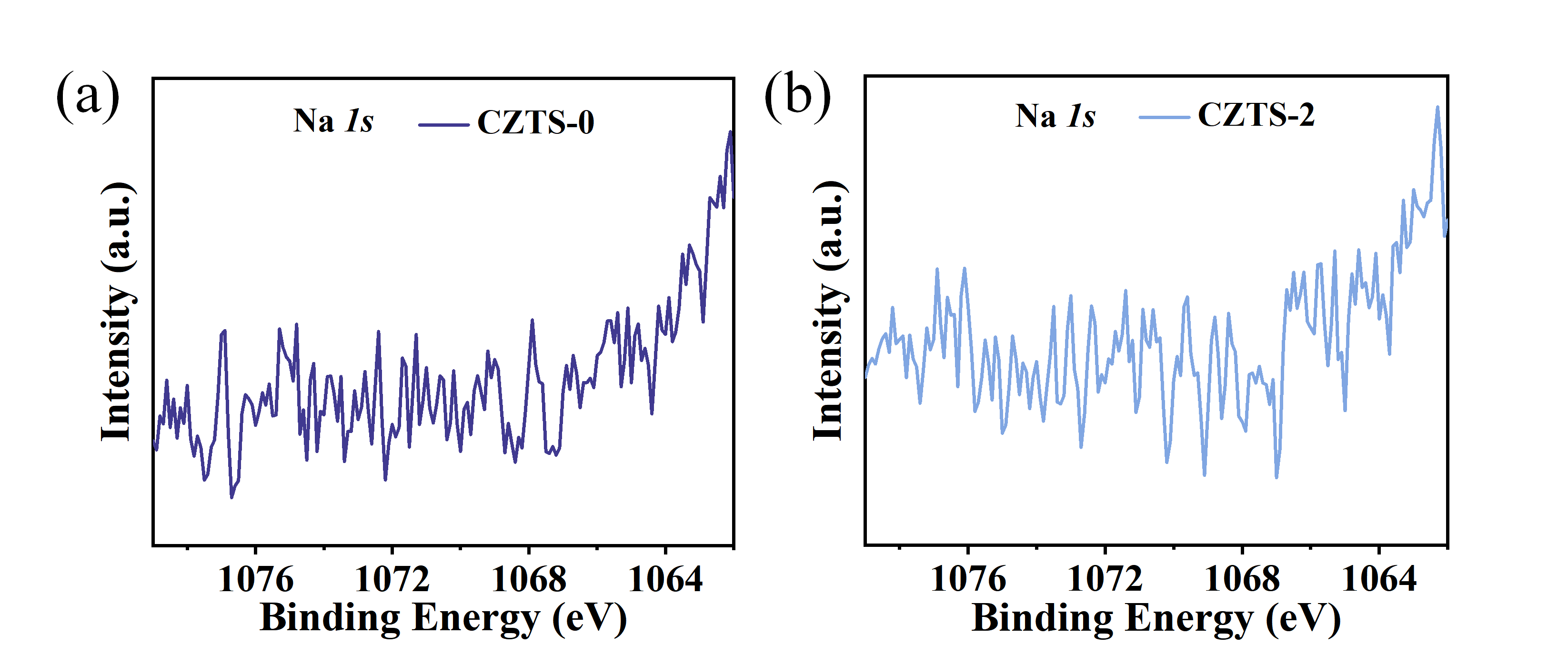


**Figure S3.** XPS analysis of Na *1s* region for (a) CZTS-0 and (b) CZTS-2 precursor films.


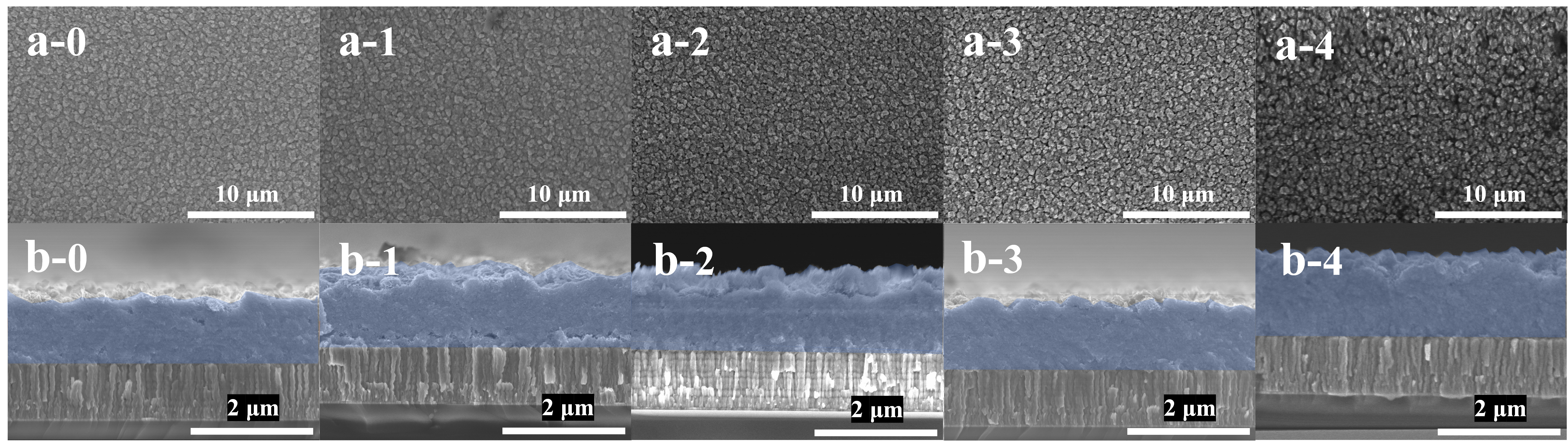


**Figure S4.** Surface (a) and cross-sectional (b) morphology of CZTS precursors with different extended sintering times. Suffixes 0, 1, 2, 3, 4 denote extended sintering times in minutes beyond the base duration.


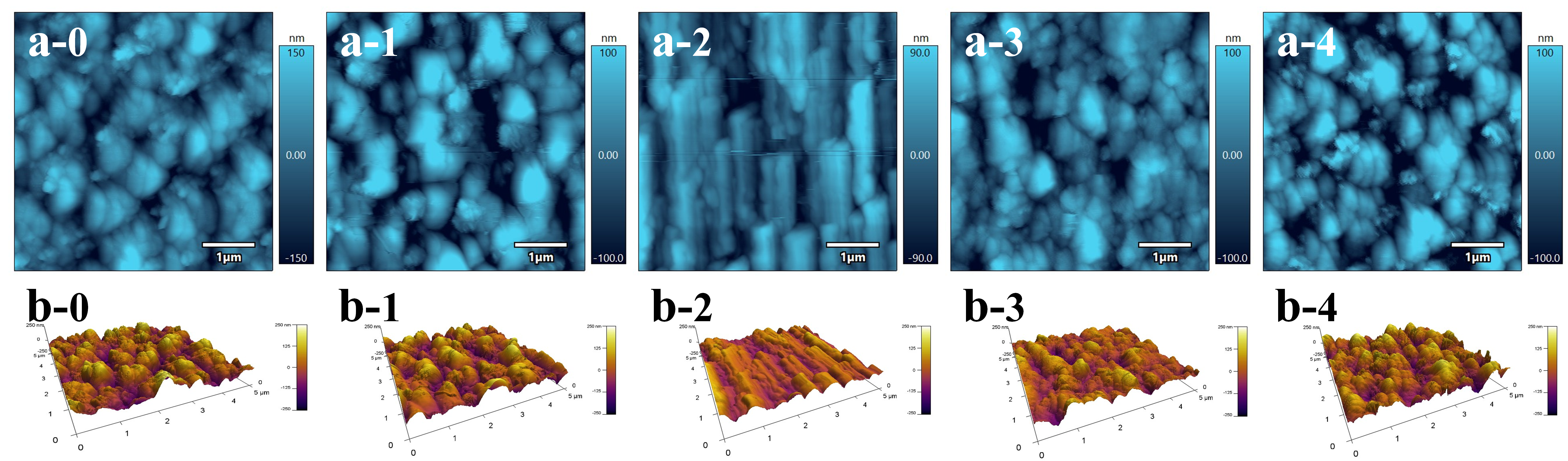


**Figure S5.** The roughness (a) and 3D profiles (b) of CZTS precursors with different extended sintering times, as characterized by AFM.


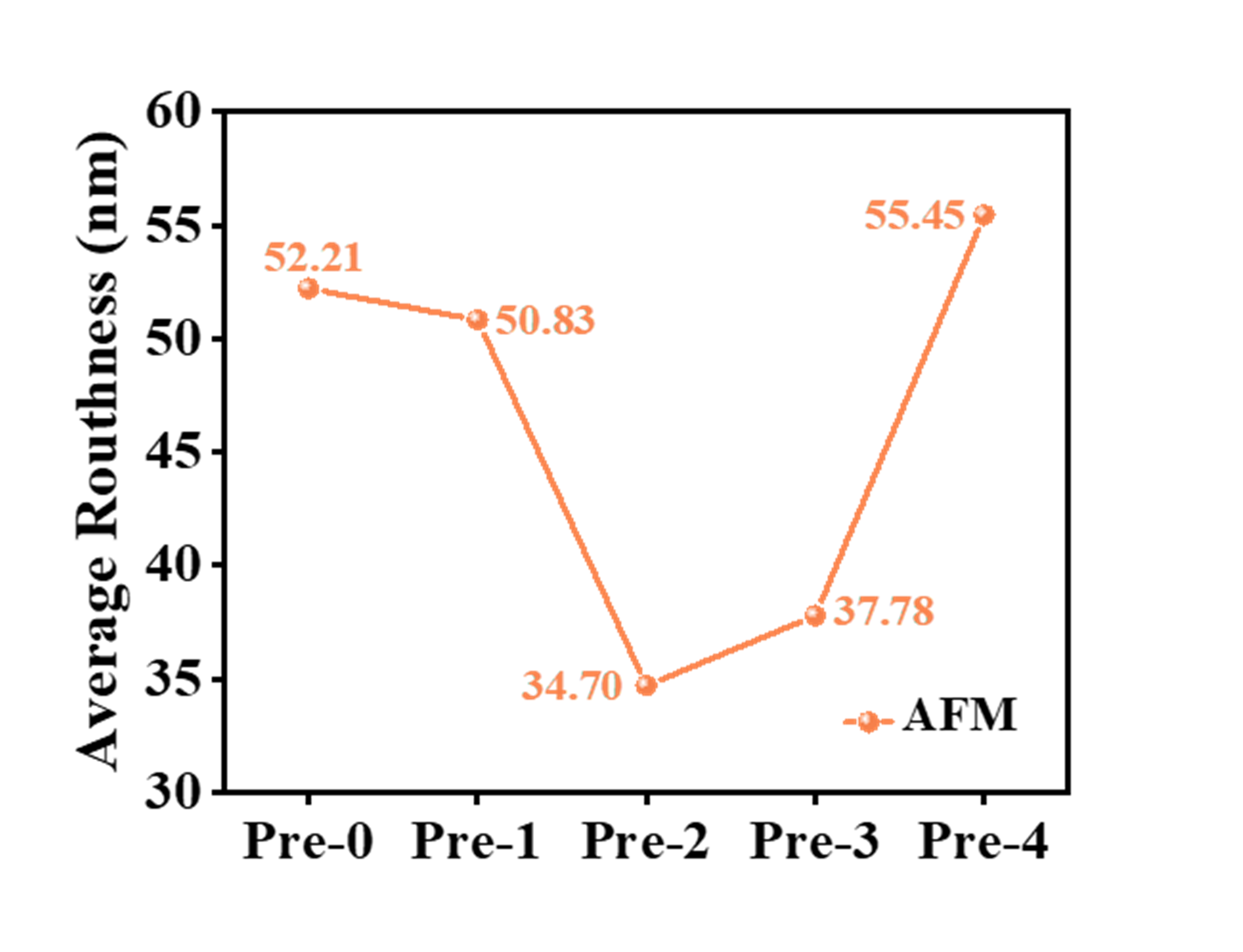


**Figure S6.** Trend of Average Roughness of CZTS precursors with different extended sintering times as depicted by AFM.


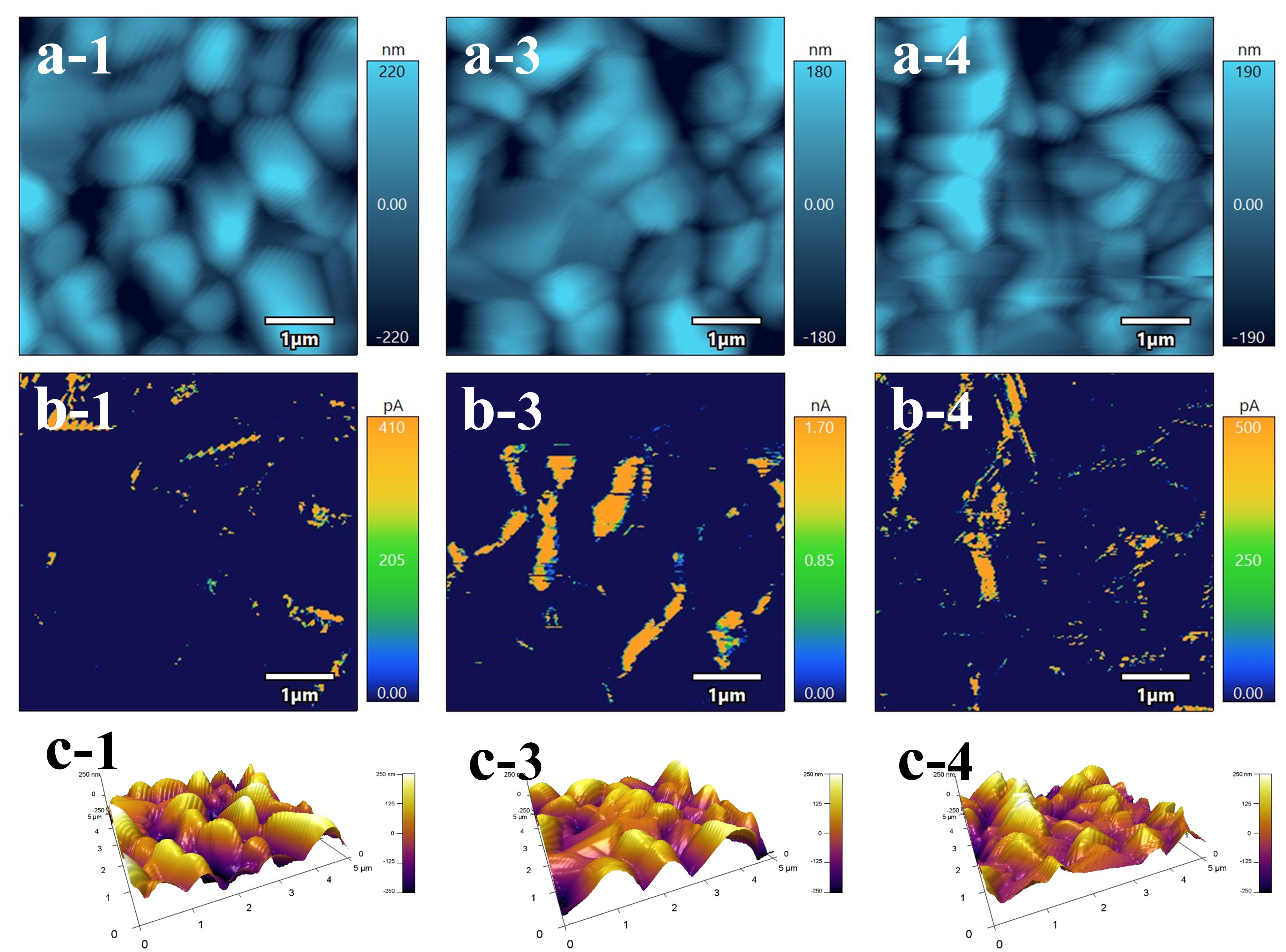


**Figure S7.** (a) Surface roughness, (b) surface current distribution, and (c) 3D profiles of CZTSSe precursors with different extended sintering times.


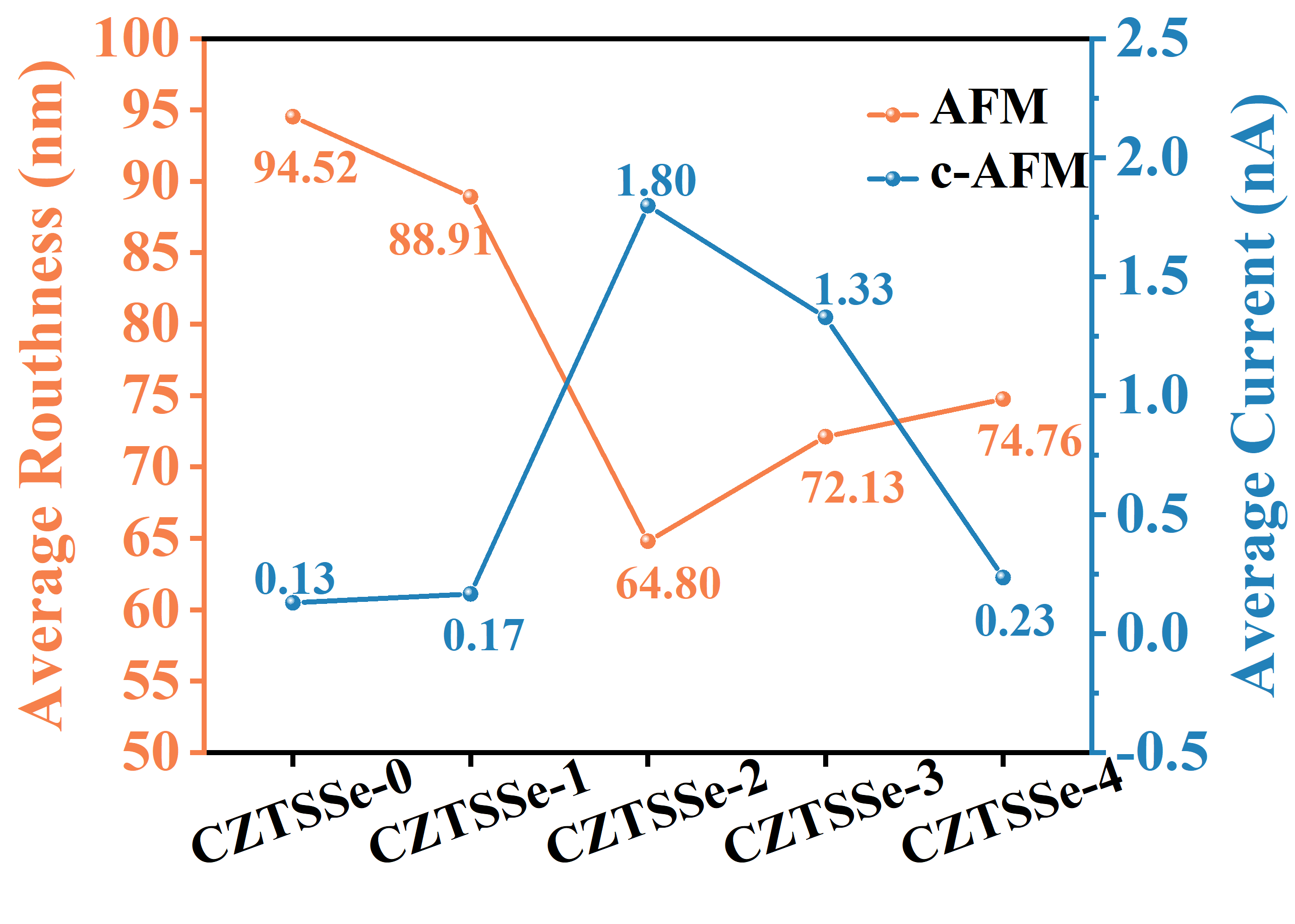


**Figure S8.** Trend of Average Roughness and surface current of CZTS precursors with different extended sintering times as depicted by AFM.


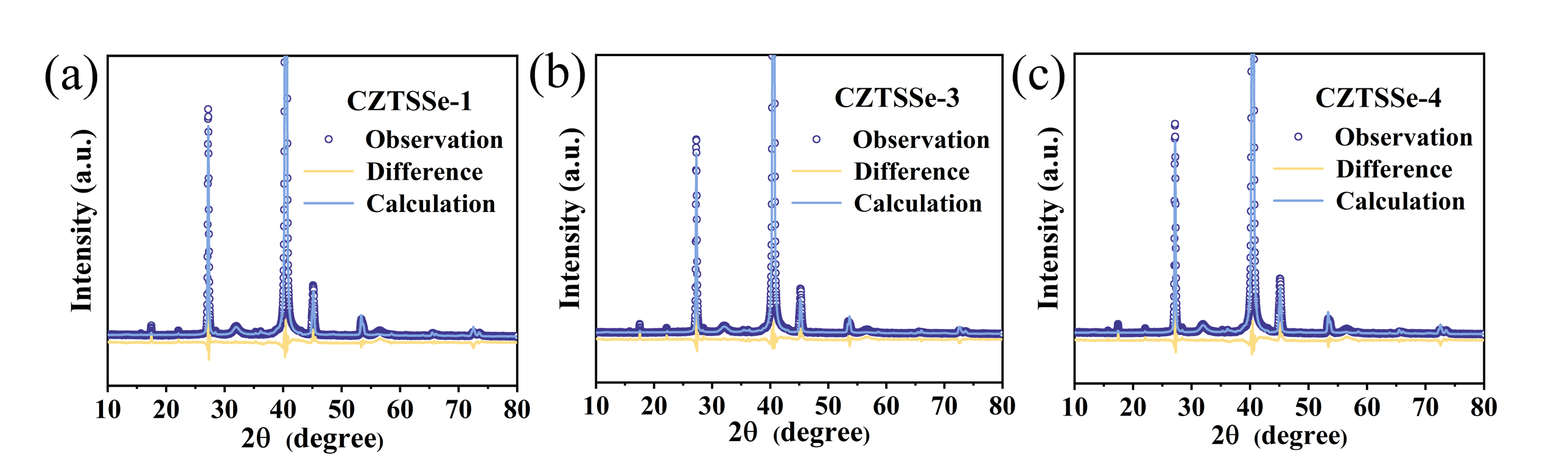


**Figure S9.** Refinement of XRD patterns for CZTSSe films with different extended sintering times.

**Table S2.** Refinement results of XRD data for CZTSSe with different extended sintering times.

| Sample | A  [Å] | Grain size  [nm] | *R_exp_* | *R_wp_* | *R_p_* | *GOF* |
| --- | --- | --- | --- | --- | --- | --- |
| CZTSSe-1 | 5.70 | 41.41 | 2.91 | 11.36 | 6.68 | 3.90 |
| CZTSSe-3 | 5.70 | 42.16 | 3.09 | 11.86 | 6.93 | 3.84 |
| CZTSSe-4 | 5.70 | 41.07 | 2.88 | 11.26 | 6.63 | 3.91 |

**
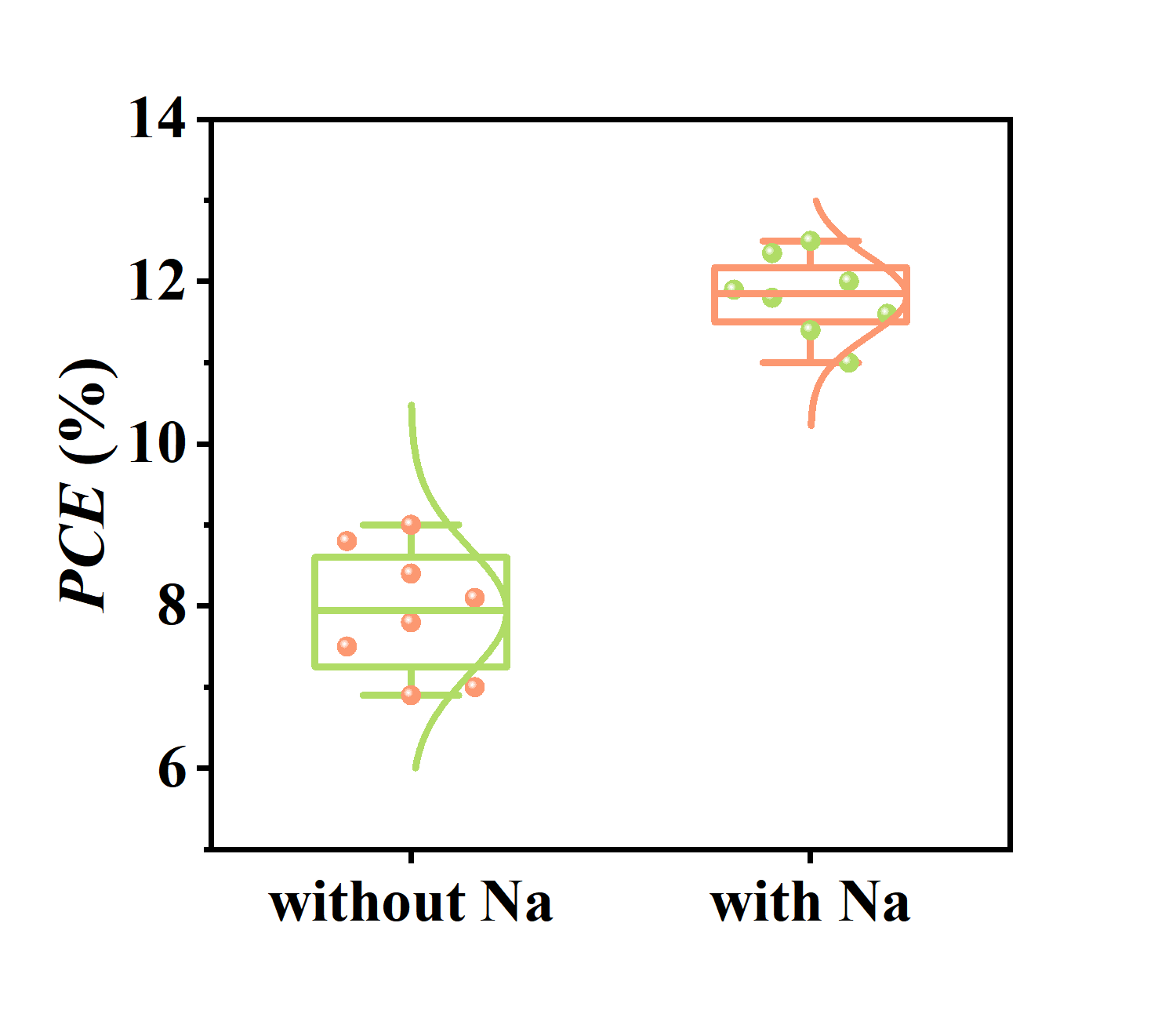
**

**Figure S10.** Efficiency statistics of CZTSSe solar cells fabricated on quartz glass (Na-free) and soda-lime glass (Na-containing) substrates.

**Table S3.** Detailed photovoltaic parameters of CZTSSe solar cells on quartz glass (Na-free) and soda-lime glass (Na-containing) substrates.

| Device | *V_OC_*  [mV] | *J_SC_*  [mA/cm^2^] | *FF*  [%] | *PCE*  [%] |
| --- | --- | --- | --- | --- |
| With Na | 499.11 | 33.53 | 70.58 | 11.81 |
| Without Na | 468.01 | 31.35 | 58.26 | 8.55 |


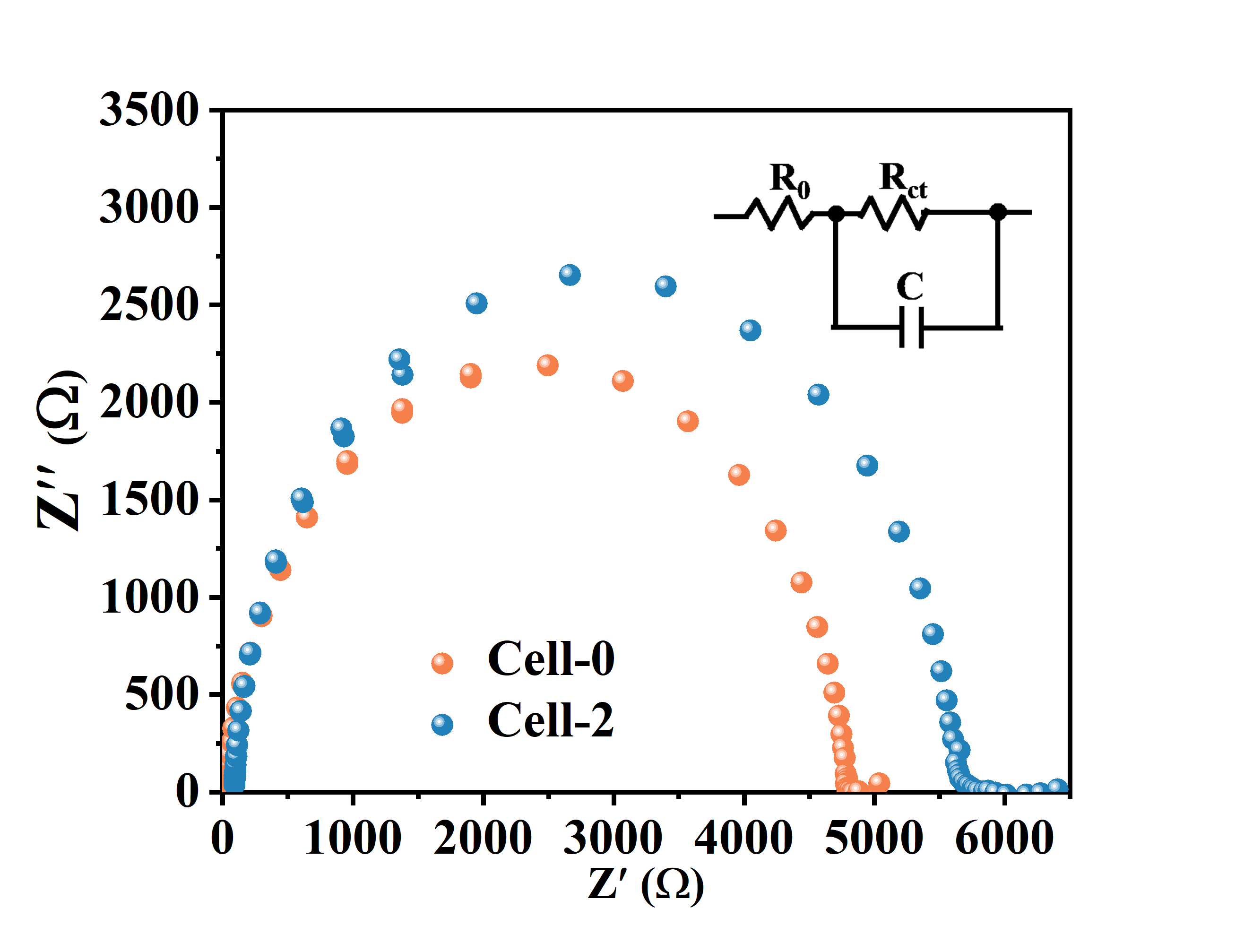


**Figure S11.** EIS Nyquist plots for the Cell-0 and Cell-2 devices.
